# Co-Administration of Molecular Adjuvants Expressing NF-Kappa B Subunit p65/RelA or Type-1 Transactivator T-bet Enhance Antigen Specific DNA Vaccine-Induced Immunity

**DOI:** 10.3390/vaccines2020196

**Published:** 2014-03-25

**Authors:** Devon J. Shedlock, Colleen Tingey, Lavanya Mahadevan, Natalie Hutnick, Emma L. Reuschel, Sagar Kudchodkar, Seleeke Flingai, Jenny Yan, Joseph J. Kim, Kenneth E. Ugen, David B. Weiner, Kar Muthumani

**Affiliations:** 1 Department of Pathology & Laboratory Medicine, Perelman School of Medicine, University of Pennsylvania, Philadelphia, PA 19104, USA; E-Mails: shedlock@mail.med.upenn.edu (D.J.S.); colleen.tingey@gmail.com (C.T.); swaanya@gmail.com (L.M.); nhutnick@gmail.com (N.H.); emmareuschel@gmail.com (E.L.R.); sagarkud@gmail.com (S.K.); seleeke@mail.med.upenn.edu (S.F.); jenyan@sas.upenn.edu (J.Y.); dbweiner@mail.med.upenn.edu (D.B.W.); 2 Inovio Pharmaceuticals Inc., 1787 Sentry Parkway West, Building 18, Suite 400, Blue Bell, PA 191422, USA; E-Mail: kim@inovio.com; 3 Department of Molecular Medicine, University of South Florida Morsani College of Medicine, Tampa, FL 33612, USA; E-Mail: kugen@health.usf.edu

**Keywords:** DNA vaccine, transcription factors, adjuvant-enhanced immunity, T cell immunity, antibody responses

## Abstract

DNA vaccine-induced immunity can be enhanced by the co-delivery of synthetic gene-encoding molecular adjuvants. Many of these adjuvants have included cytokines, chemokines or co-stimulatory molecules that have been demonstrated to enhance vaccine-induced immunity by increasing the magnitude or type of immune responses and/or protective efficacy. In this way, through the use of adjuvants, immune responses can be highly customizable and functionally tailored for optimal efficacy against pathogen specific (*i.e.*, infectious agent) or non-pathogen (*i.e.*, cancer) antigens. In the novel study presented here, we examined the use of cellular transcription factors as molecular adjuvants. Specifically the co-delivery of (a) RelA, a subunit of the NF-κB transcription complex or (b) T-bet, a T_h_1-specific T box transcription factor, along with a prototypical DNA vaccine expressing HIV-1 proteins was evaluated. As well, all of the vaccines and adjuvants were administered to mice using *in vivo* electroporation (EP), a technology demonstrated to dramatically increase plasmid DNA transfection and subsequent transgene expression with concomitant enhancement of vaccine induced immune responses. As such, this study demonstrated that co-delivery of either adjuvant resulted in enhanced T and B cell responses, specifically characterized by increased T cell numbers, IFN-γ production, as well as enhanced antibody responses. This study demonstrates the use of cellular transcription factors as adjuvants for enhancing DNA vaccine-induced immunity.

## 1. Introduction

DNA vaccination has once again became elevated to the forefront of efforts aimed at developing vaccines against challenging infectious diseases including HIV/AIDS, emerging strains of influenza as well as SARS. As well, it has a reemerging role as a delivery method for tumor immunotherapy [1,2,3,4,5,6,7,8]. While “first-generation” DNA vaccines were poorly immunogenic, recent technological advances have dramatically improved their ability to drive immunity, including cellular based responses, in preclinical immunogenicity and efficacy studies [9,10,11,12,13,14] as well as in clinical trials [15,16,17,18]. The transfection rate of plasmid DNA and subsequent expression of their encoded antigens (Ages) are significantly enhanced when highly-concentrated plasmid vaccine formulations are delivered through *in vivo* electroporation (EP), a technology using brief square-wave electric pulses at the vaccination site to facilitate entry and expression of DNA plasmids into transiently permeabilized cells resulting in improved immunogenicity and efficacy of the vaccines [19]. In theory, a cocktail of plasmids could be assembled for directing a highly-specialized immune response against any number of variable antigens (Ag), which, in turn, could induce a more robust and efficacious immune response. In addition, “consensus-engineering” of the Ag amino acid sequences has been effectively used to help bias vaccine-induced immunity towards particular divergent, circulating, or virulent strains such as enhancing protection among divergent strains of HIV and influenza virus [20,21]. Due, in part, to these technological developments, immunization regimens including these “enhanced” DNA (E-DNA) vaccines are extremely customizable and highly versatile.

Immunity can be further directed by co-delivery of the vaccine with plasmid-based molecular adjuvants encoding species-specific immunomodulatory proteins. These have typically included cytokines, chemokines, and surface expressed co-stimulatory molecules [18,22,23,24,25,26,27,28,29,30,31,32,33,34]. Such a gene adjuvant approach substantially enhanced immune potency in numerous vaccine studies [16,18,29,35,36]. As a candidate for molecular adjuvant development, transcription factors regulate the gene expression of numerous inflammatory factors and promote activation and maturation of the adaptive immune response [37,38,39]. An established pro-inflammatory mediator is the NF-kappa B protein complex which regulates the expression of cytokines (TNF-α, IL-1β, IL-6, IL-2, *etc*.), induces DC maturation (characterized by upregulation of CD80, CD86 and CD40), and promotes cell survival by the regulation of Bcl-XL and IAPs, and protecting against TNF-α induced cell death [40,41,42]. This transcription factor consists of five members including p65 (RelA), c-Rel, RelB, p50 (NF-κB1) and p52 (NF-κB2) [42]. Importantly, RelA is a vital component in inflammation and cell survival, possesses transcriptional activating capabilities, and can potently activate kappa B-dependent transcription. Upregulation of this subunit may regulate the gene expression of multiple inflammatory and survival factors that may lead to improved adaptive immunity [43,44,45,46]. 

An additional candidate for development as a molecular adjuvant is T-bet, a T helper 1 (T_h_1)-specific transcription factor, which would ideally help to promote the induction of T_h_1-type immunity. This T-box family member regulates lineage commitment in CD4 Th cells by directly activating transcription of the IFN-γ gene. It also exhibits the property of redirecting committed T_h _2 populations to a T_h_1 phenotype [47,48]. The importance of T-bet in T_h_1 immunity has been most clearly illustrated and reported in cases where CD4^+^ T cells lacking T-bet are severely impaired in their ability to produce IFN-γ, yet secrete elevated levels of the opposing Th2 subset cytokines, IL-4 and IL-5. Co-expression of this protein along with a vaccine against tuberculosis has been demonstrated to increase IgG2a antibody (Ab) responses as well as the production of IFN-γ and IL-2 [47]. Thus, a T-bet-expressing molecular adjuvant delivered by E-DNA vaccination would ideally enhance the magnitude and type of immunity induced by immunization. Together, RelA and T-bet present attractive candidates, as molecular adjuvants, for the enhancement of immunity following E-DNA vaccination.

In this study, we investigated the ability of molecular adjuvants expressing transcription factors RelA or T-bet, as developed herein, to enhance in mice, immunity of an E-DNA vaccine encoding either HIV-1 Env or Gag. Their potential ability to modify immunity was then assessed. Co-administration of either pRelA or pTbet in conjunction with the pEnv or pGag vaccine significantly increased T cell immunity, as measured by INF-γ production by ELISpot and proliferation. As well, B-cell/antibody levels were enhanced as indicated by an increase in B-cell numbers as well as antigen specific antibody titers. Consistent with these findings, the total amount of antigen specific IgG in serum was increased following the co-administration of plasmids expressing the transcription factors. This study builds on recent successes in demonstrating the potency of E-DNA vaccination and suggests that transcription factors may serve as an effective adjuvant to increase vaccine-induced immunity.

## 2. Experimental

### 2.1. Plasmid Vaccine Constructs

The pRelA plasmid DNA constructs encode the full-length mouse NF-κB subunit p65/RelA (GenBank #TF65_MOUSE) and Type-1 transactivator T-bet (GenBank #TBX21_MOUSE), respectively. In addition, the Ig heavy chain epsilon-1 signal peptide (GenBank#AAB59424) was fused to the *N*-terminus of each sequence, replacing the *N*-terminal methionine, which facilitates expression [11,49]. Each gene was genetically optimized for expression in mice, including codon- and RNA-optimization, among other proprietary modifications for enhancing protein expression (GenScript, Piscataway, NJ, USA). The optimized genes were then sub-cloned into modified pVax1 mammalian expression vectors (Invitrogen, Carlsbad, CA, USA) under the control of the cytomegalovirus immediate-early (CMV) promoter. These reagents were then used as the molecular adjuvants in this study. The pGag [50] and pEnv [51] plasmids, expressing the HIV-1 proteins Gag and Env respectively, have been previously described.

### 2.2. Transfections and Western Blot Analysis

Human Embryonic Kidney (HEK) 293T cells were maintained in Dulbecco’s modified Eagle medium (Life Technologies, Grand Island, NY, USA), supplemented with 10% heat-inactivated fetal calf serum (FCS), 100 IU of penicillin per mL, 100 μg of streptomycin per mL and 2 mM l-glutamine [9]. Briefly, cells were transfected using TurboFection 8.0 (OriGene, Rockville, MD, USA) per the manufacturer’s protocol and subsequently incubated for 24–48 h. Cells were harvested with ice cold PBS, centrifuged and washed, and then pelleted for Western immunoblot analysis [52]. Nuclear extracts (10^7^ cells) were made according to the method of Muthumani *et al.* [52]. The nuclear proteins from the transfected cells were then dissolved in 20 mM Hepes (pH 7.9) containing 0.4 M NaCl, 1 mM EDTA, 1 mM EGTA, 1 mM DTT, 1 mM PMSF and a cocktail of protease inhibitors (Promega Corp, Madison, WI, USA). The protein concentration of each extract was measured by the Bio-Rad protein assay kit (Bio-Rad, Hercules, CA, USA), and extracts were stored in aliquots at −70 °C until used. Standard western blotting analysis was performed. Cells were treated with protein lysis buffer (0.01 M Tris-HCl buffer pH 7.4, containing 1% Triton X-100, 1% sodium deoxycholate, 0.1% SDS) supplemented with protease inhibitors (Protease Inhibitor Cocktail tablets; Roche, Indianapolis, IN, USA). Proteins in lysates were then separated using 12% SDS-PAGE [53]. Protein-specific detection antibodies for RelA and T-bet (Cell Signaling Technology, Danvers, MA, USA) were incubated with the blots and expression visualized using the enhanced chemiluminescence (ECL) Western blot detection system (GE Healthcare, Piscataway, NJ, USA).

### 2.3. Confirmation of Transcription Activity of RelA/p65 and T-Bet by Luciferase Reporter Assay and IFN-Gamma Production

A RelA/p65 expressing vector, which co-expresses luciferase (pNF-κB-Luc) was used to confirm the functionality of RelA/p65, which is necessary before it being used the “adjuvanted” vaccine study. The luciferase reporter assay was performed as described previously [52,54,55]. Briefly, 293T cells (10^5^ cells/well) were seeded in a 96-well plate for 24 h. The cells were then transfected with the RelA/p65 Luc expressing plasmid followed by incubation for 6 h. After incubation, the cell culture medium was removed and replaced with fresh medium. Two days post transfection cells were treated with 20 ng/mL of recombinant TNF-α for 6 h followed by measurement of luciferase activity by using Microlumat plus luminometer (LUMAT LB9501, Berthold Technologies, Oak Ridge, TN, USA). 

For confirmation of pT-bet function, the production of IFN-γ from pT-bet transfected CD4^+^ T cells was measured. The impetus for measurement of IFN-γ is based on previously published studies that demonstrated a direct correlation between T-bet and IFN-γ production [56]. Briefly in this analysis naïve CD4^+^ T cells, isolated from the spleens of Balb/C mice, were purified using a CD4^+^ T cell isolation kit (Miltenyibiotec, San Diego, CA, USA). These cells were maintained in RPMI media supplemented with 10% FBS, 100 U/mL penicillin and 200 µg/mL streptomycin and subsequently transfected with pT-bet or pVax1 as a negative control. Two days post-transfection, cells were stimulated overnight with anti-CD3 plus anti-CD28 Abs (1 µg/mL). IFN-γ levels in the supernatants collected from the cultured CD4^+^ T cells were subsequently measured by a standard ELISA [36].

### 2.4. Animals and Vaccination Regimen

Adult female BALB/cJ (H-2^d^) mice were purchased from The Jackson Laboratory (Bar Harbor, ME, USA). All animal experimentation was conducted according to University of Pennsylvania (UPENN) IACUC approved protocols and performed in accordance with recommendations in the Guide for the Care and Use of Laboratory Animals of NIH. UPENN complies with NIH policy as stated in the Animal Welfare Act, and all other applicable federal, state and local laws. Mice were immunized intramuscularly (i.m.) by needle injection into the left-thigh quadriceps muscle with 25 µg of plasmid resuspended in 25 µL of PBS. Vaccinations were immediately followed by EP, at the same site, and repeated at a two-week interval. For EP mediate delivery, a three-pronged CELLECTRA^®^ adaptive constant current Minimally Invasive Device (MID) was used, supplied by Inovio Pharmaceuticals, Inc. (Blue Bell, PA, USA). Specifically, square-wave pulses were delivered through a triangular 3-electrode array (inserted 2 mm intradermally) consisting of 26-gauge solid stainless steel electrodes and two constant-current pulses of 0.1 Amps were delivered for 52 msec/pulse separated by a 1 s delay. During the vaccination/molecular adjuvant administration regimen, and through the termination for the study, all mice were monitored every 3 days for the development of potential adverse effects.

### 2.5. Splenocyte, T Cell Isolation and Cytokine Quantitation

Spleens were harvested 7–8 days following the third immunization as previously described [12]. Briefly, spleens were placed in RPMI 1640 medium (Mediatech, Manassas, VA, USA) supplemented with 10% FBS, 1X Antibiotic-Antimycotic (Life Technologies, Grand Island, NY, USA), and 1× β-ME (Life Technologies, Grand Island, NY, USA). Splenocytes were isolated by mechanical disruption of the spleen using a Stomacher machine (Seward Laboratory Systems, Bohemia, NY, USA), and the resulting product was filtered using a 40 μm cell strainer (BD Biosciences, San Jose, CA, USA). The cells were then treated for 5 min with ACK lysis buffer (Lonza, Walkersville, MD, USA) for lysis of RBCs, washed in PBS, and then resuspended in RPMI medium for use in the ELISPOT assay. CD4 naïve T cells were purified from the spleens using a naïve CD4^+^ T cell isolation kit (Miltenyi Biotec, Auburn, CA, USA). These cells were maintained in RPMI medium supplemented with 10% FBS, 100 U/mL penicillin, 200 μg/mL streptomycin, and stimulated with anti-CD3 plus anti-CD28 (1 μg/mL each). Upon stimulation with anti-CD3 plus anti-CD28 antibodies, cytokine production levels in the culture supernatants of cultured cells were examined by enzyme-linked immunosorbent assay (ELISA) as described previously [3,13].

### 2.6. ELISPOT Analysis

The standard IFN-γ ELISPOT assay used in this study has been previously described [9,11,12]. Briefly, 96-well plates (Millipore, Billerica, MA, USA) were coated with anti-mouse IFN-γ capture antibody and incubated for 24 h at 4 °C (R&D Systems, Minneapolis, MN, USA). The following day, plates were washed with PBS and then incubated for 2 h with blocking buffer (1% BSA and 5% sucrose in PBS). CD4^+^ or CD8^+^ T cells (5 × 10^5^ cells/well plated in triplicate) were MACS-purified (Miltenyibiotec, San Diego, CA, USA) from splenocytes and subsequently stimulated with HIV-1 Gag (consensus subtype B) or Env (subtype B (MN)) peptides (15-mers overlapping by 11 amino acids, spanning the lengths of their respective protein (NIH AIDS Reagent Program, Bethesda, MD, USA). After 18–24 h of stimulation overnight at 37 °C in 5% CO_2_, the plates were washed in PBS and subsequently incubated for an additional 24 h at 4 °C with biotinylated anti-mouse IFN-γ monoclonal antibody (mAb) purchased from R&D Systems (Minneapolis, MN, USA). The plates were then washed again in PBS, and streptavidin-alkaline phosphatase (MabTech, Nacka Strand, Sweden) was added to each well and incubated for 2 h at RT. Lastly, the plates were washed again in PBS followed by incubation with BCIP/NBT Plus substrate (MabTech, Cincinnati, OH, USA) for 5–30 min. Upon completion of spot development based on visual inspection, the plate was rinsed with distilled water and then dried overnight at RT. Spots were enumerated using an automated ELISPOT reader (Cellular Technology, Shaker Heights, OH, USA).

### 2.7. T Cell Proliferation Assay

Proliferative responses were measured *in vitro* by incubating 10^5^ splenocytes in culture medium per well in 96-well U-bottom plates in the presence of serial dilutions (5, 1, and 0.1 μg/mL) of recombinant HIV-1 IIIB pr55 (Gag) (NIH AIDS Reagent Program, Bethesda, MD) or HIV-1 MN IIIB gp160 (Env) (Protein Sciences, Meriden, CT, USA) and incubated at 37 °C with 5% CO_2_. Incorporation of tritiated (^3^H)-thymidine was measured by pulsing with 1 μCi/well of (^3^H)-thymidine during a 0–24 h time period as described previously [57]. The plate was then harvested and incorporated ^3^H-thymidine was measured in a Beta plate reader (Wallac, Waltham, MA, USA). The proliferative response is expressed as a stimulation index (SI), calculated by dividing the mean cpm (counts per minute) of Ag-stimulated wells by the mean cpm of non-stimulated wells.

### 2.8. ELISA

Sera from vaccinated mice harvested 7 days following the third vaccination were tested for antibody responses against recombinant HIV-1 Env (NIH AIDS Reagent Program) by ELISA. Briefly, 96-well ELISA plates were coated with recombinant HIV-1 Env protein (Protein Sciences) and incubated at 4 °C and washed subsequently with PBS and 0.1% Tween-20. Plates were then blocked for 2 h with PBS and 0.2% Tween-20. After removal of the blocking solution, 100 μL of the pre-diluted (1:50, 1:100, 1:500, 1:1000) mouse serum was added and incubated for 1 h. Plates were then washed four times and incubated with a peroxidase-coupled anti-mouse IgG mAb (Sigma-Aldrich, St. Louis, MO, USA). Lastly, plates were washed again followed by addition of 200 μL of substrate solution (R&D Systems, Minneapolis, MN, USA) per well. The optical density at (OD_405_ nm) was subsequently measured after a 15 min incubation. All assays were performed in triplicate.

### 2.9. Flow Cytometry

Muscle tissues (*i.e.*, from the site of injection/vaccination) were removed aseptically, rinsed in Hanks’ balanced salt solution (Life Technologies, Grand Island, NY, USA), minced into approximately 1 × 2-mm squares, and digested in 20 mL of collagenase A (1 mg/mL, Life Technologies, Grand Island, NY, USA) at 37 °C for 45 min, with occasional agitation. The cellular digest was filtered through a sterile 31 μm nylon mesh, centrifuged at 400 g for 10 min, and washed twice in 10% FCS-DMEM. The cell pellet was then resuspended in 4 mL of 10% FCS-DMEM.

For flow cytometric analysis, 10^6^ cells from the immunized mice cells were washed in suspension with ice-cold buffer A (PBS/0.1% BSA/0.01% NaN3) and incubated for 20 min at 4 °C with 50 μL of a 1:100 diluted fluorescent-labeled specific antibodies using methods described previously [58]. The fluorescently conjugated Abs utilized were FITC-CD11c, PE-CD4, PE-Cy7-CD45R (B220) (eBioscience, San Diego, CA, USA), Alexa Fluor-750-CD8α, and PerCP-Cy5.5-CD11b (BD Biosciences, San Jose, CA, USA). Cells were washed twice and immediately analyzed on a flow cytometer (Becton Dickinson FACS, San Jose, CA, USA). All incubations and washes were performed at 4 °C with ice-cold buffer A. Cells were gated on singlets and live cells. The flow cytometric data were analyzed using FlowJo software (Tree Star, Ashland, OR, USA).

### 2.10. Statistical Analysis

Group analyses were completed by a matched, two-tailed, unpaired *t*-test with all values are presented as mean ± SEM. Mann-Whitney analysis was used to determine statistical differences. All data were analyzed using GraphPad Prism5 Software [59]. Statistically significant differences between groups were defined as * *p* < 0.1, ** *p* < 0.01, *** *p* < 0.001, and **** *p* < 0.0001. 

## 3. Results

### 3.1. Adjuvant Construction and Expression

**Figure 1:**
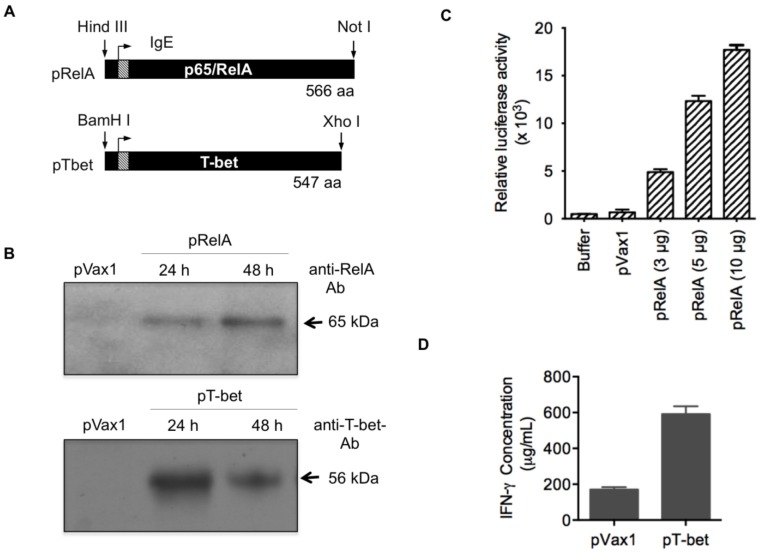
Molecular adjuvant construction and expression. (**A**) Mouse RelA or T-bet primary sequences were genetically optimized, synthesized, and then subcloned into modified pVax1 expression vectors. Optimization entailed inclusion of a IgE leader peptide (IgE), preceded by a Kozak sequence, fused at the *N*-terminus. The figure indicates the restrictions enzymes used for subcloning, the translation initiation site (forward arrow), IgE leader peptide (IgE; hatched bar), protein length (aa), and transgenes (black with white lettering); (**B**) Protein expression from the nuclear extract was analyzed by Western immunoblotting following transfection of HEK 293T cells with pRelA, pTbet, or empty vector control (pVax1). The relative size (kDa) of the proteins are determined by detection analysis using protein-specific Abs as indicated; (**C**) Over expression of RelA potently induces κB dependent transcription. HeLa cells were transiently transfected with a NF-κB-dependent luciferase reporter gene together with expression vectors encoding RelA/p65. The cotransfected cells were subsequently grown for 48 h, and the luciferase activity was determined as described in the Materials and Methods; (**D**). Overexpression of T-bet stimulates production of IFN-γ: Naive CD4 T cells were transfected with either pT-bet or pVax1 and stimulated with anti-CD3 plus anti-CD28 followed the measurement of IFN-γ production by enzyme-linked immunosorbent assay (ELISA) as described Materials and Methods. IFN-γ levels are expressed as μg/mL

The pRelA and pTbet plasmids encode the full-length mouse NF-kappa B subunit p65/RelA and Type-1 transactivator T-bet, respectively. Each was genetically optimized, synthesized, and subcloned into modified pVax1 mammalian expression vectors (Figure 1A). To test for expression of these plasmids, HEK 293T cells were transfected with each and protein production was assessed by standard Western immunoblotting. An approximately 65 kDa protein corresponding to RelA was detected, using a specific Ab, in cell lysates harvested both 24 h and 48 h post-transfection (Figure 1B). Likewise, T-bet was detected as an approximately 56 kDa protein using an anti-T-bet Ab. Binding was specific for their respective proteins since neither bound to lysates from cells transfected with empty vector control plasmid pVax1. These data demonstrate that each of the molecular adjuvants expresses their respective encoded proteins upon *in vitro* transfection of HEK 293T cells. Further, IκB-dependent transcription was accessed in the HeLa cells luciferase expressing cell system (Figure 1C) to confirm the activation of RelA (p65). An increase in RelA expression as measured by relative luciferase activity was observed in a dose dependent manner. That is, increasing the plasmid from 3 μg to 5 μg or 10 μg resulted in an increase in the relative luciferase activity approximately 1.5 or 2.5 fold. T-bet expression correlates with IFN-γ expression in T cell and NK cells [60] and therefore in this assay IFN-γ serves as surrogate for the functional expression of T-bet (Figure 1D). 

### 3.2. Enhanced Cellular Immunity

**Figure 2:**
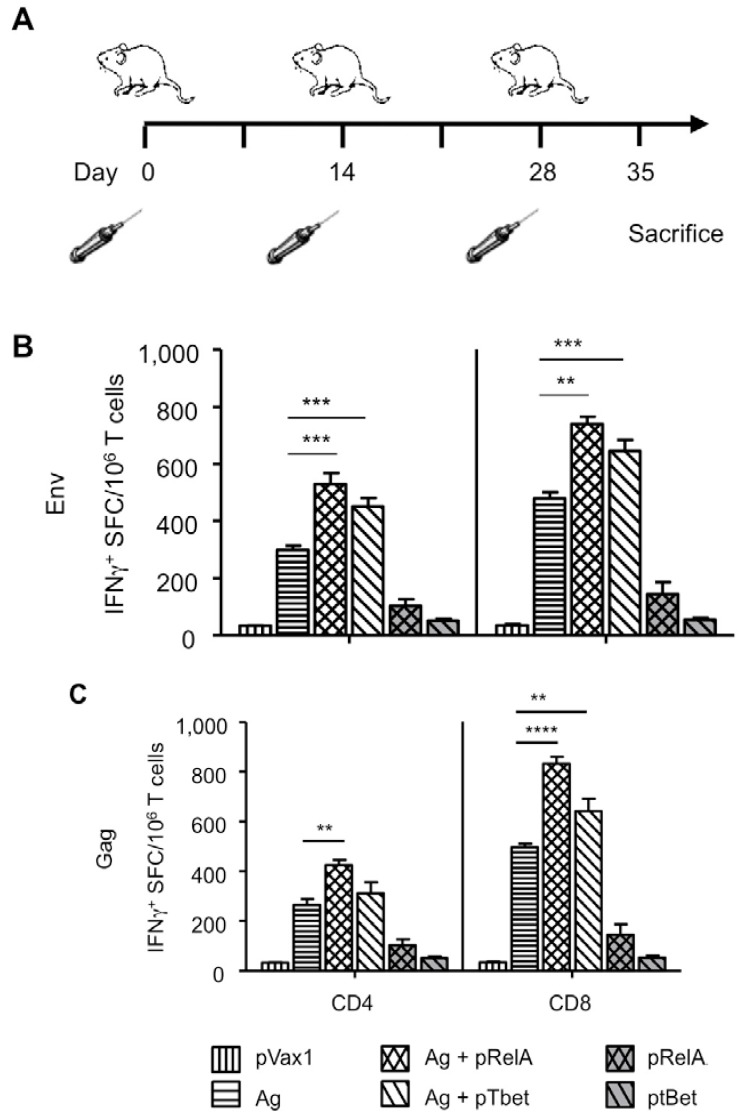
Transcription factor adjuvants enhance antigen specific DNA vaccine induced T cell immunity. (**A**) Balb/C mice (*n= 4*/group) were vaccinated three times at two week intervals with HIV-1 pGag or pEnv alone, pGag or pEnv with co delivery of either pRelA or pTbet. Other control groups were pRelA or pTbet alone, or a pVax1 control. T cell responses (CD8^+^ and CD4^+^) were analyzed by IFN-γ ELISPOT one week following the third immunization and results for IFN-γ+ spot forming cells (SFC) per 10^6^ MACS-purified T cells are indicated following re-stimulation with subtype B HIV-1 Env (**B**) or Gag (**C**) peptide pools. Samples were performed in triplicate, error bars represent SEM, and statistically significant values are shown; ** *p* < 0.01, *** *p* < 0.001 and **** *p* < 0.0001, referring to comparison between the indicated vaccination groups provided in the graph. Experiments were performed twice independently with similar results.

The contribution of pRelA and pTbet, in terms of enhancing vaccine-induced immunity, was then assessed. Balb/C mice (*n* = 4/group) were vaccinated three times with 25 µg of pEnv or pGag either with or without 25 µg of pRelA or pTbet, 25 µg of pRelA or pTbet alone, or with 25 µg of a control plasmid (pVax1; Figure 2). The vaccines and adjuvants were delivered in 25 µL of PBS by *in vivo* EP. Animals were sacrificed on day 35, (*i.e.*, seven days after the third vaccination) followed by isolation of splenocytes for immune analysis by IFN-γ ELISpot. In this assay, HIV-1 Env or Gag peptide pools were used for stimulation of MACS-purified CD4^+^ or CD8^+^ T cells and the IFN-γ ELISpot results are displayed in Figure 2. Both CD4^+^ and CD8^+^ T-cell responses were observed to be significantly increased in mice vaccinated with pEnv and co-administrated pRelA compared with pEnv alone. Likewise, immunization with pEnv with co-administrated pTbet compared to pEnv alone demonstrated significant increases in CD4^+^ and CD8^+^ T cell responses (Figure 2B). 

To confirm the enhancing effects of these two adjuvants on T cell IFN-γ production for a different Ag, we also vaccinated animals with the HIV-1Gag either with or without pRelA or pTbet, similarly as performed above. Analogous to the pEnv group, CD4^+^ T cell responses were increased in mice immunized with pGag plus co-administrated pRelA, when compared with mice immunized with pGag alone (Figure 2C). There was an even greater enhancement of the CD8^+^ T cell response in mice vaccinated with pGag and co-administrated pRelA compared to immunization with pGag alone (Figure 2C). Further, immunization with HIV-1 Gag along with concomitant administration of pTbet demonstrated increased CD8^+^ T-cell responses when compared to immunization with pGag alone (Figure 2C). However, CD4^+^ T cell responses were not as significantly increased as observed with co-delivery of pRelA. Also, administration of either pRelA or pTbet alone did not markedly activate either CD4^+^ or CD8^+^ T cells against Gag or Env as measured by IFN-γ production. Therefore, these data demonstrate that co-administration of the transcription factor adjuvants promoted enhanced T cell responses against two separate antigens with the data suggesting that expanding the breadth of vaccine-elicited cellular immune responses was stimulated by administration of an immune adjuvant.

**Figure 3:**
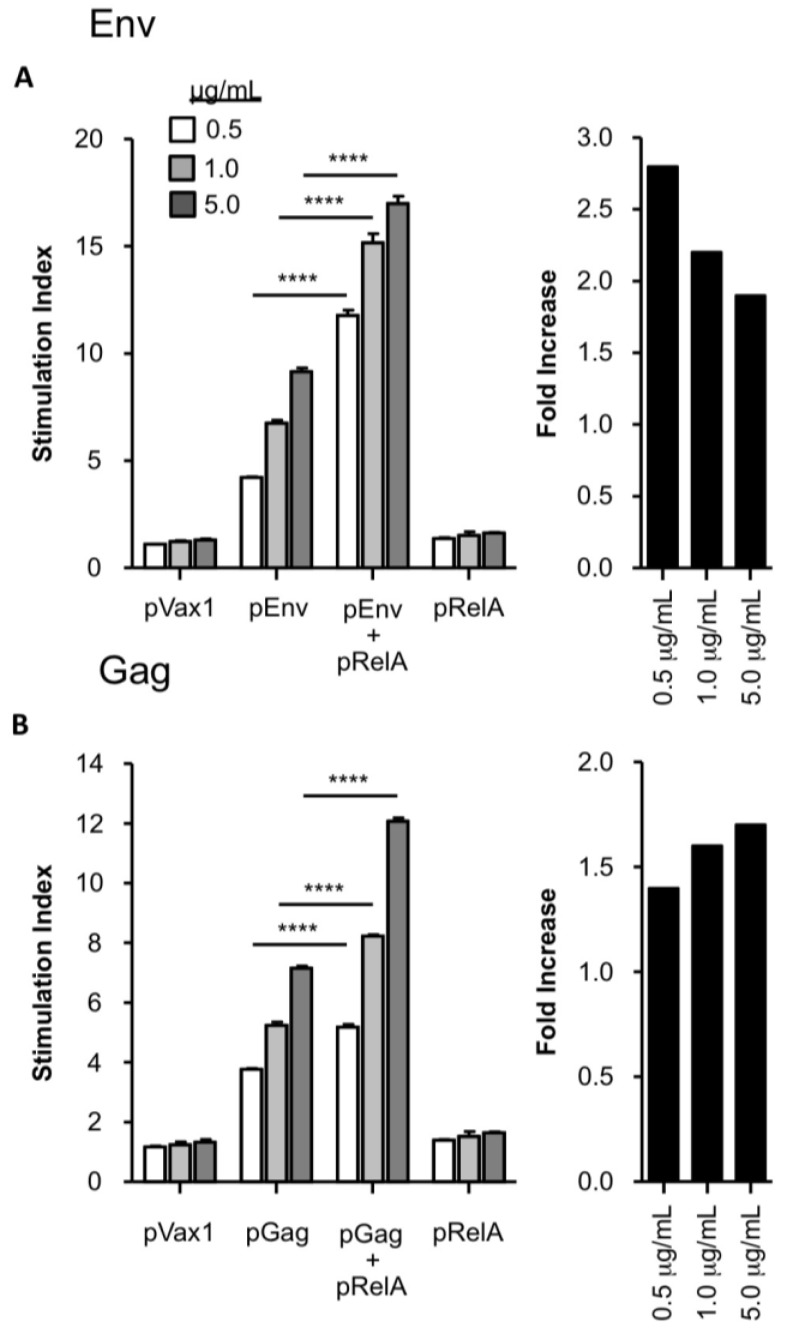
Increased T-cell proliferative potential following DNA vaccination plus co-administration of pRelA. Proliferative responses were measured seven days following the third vaccination with either pEnv or pGag alone, pEnv or pGag with pRelA molecular adjuvant, or empty vector control pVax1 alone. Splenocytes were incubated with recombinant HIV-1 Env (**A**) or Gag (**B**) at various concentrations: 0.5 (white bars), 1.0 (light gray bars), and 5.0 (dark gray bars) and subsequently pulsed with tritiated (^3^H)-thymidine for 24 h. Incorporated thymidine was expressed as a stimulation index (SI) calculated by dividing the mean cpm (counts per minute) of Ag-stimulated wells by the mean cpm of non-stimulated wells. Fold increase in SI for pRelA-adjuvanted mice are displayed for each concentration of Env (**A**, right panel) or Gag (**B**, right panel). Samples were tested in triplicate. Error bars represent the SEM, and statistically significant values are provided for the indicated group comparison shown in the graphs. **** *p* < 0.0001.

Since the RelA molecular adjuvant was observed to particularly enhance T cell responses, the proliferative potential of cells immunized in the presence or absence of pRelA was evaluated. Splenocytes from vaccinated animals were harvested at seven days following the third immunization and were then stimulated with their cognate Ag, *i.e.*, either HIV-1 Env or Gag (Figure 3). In pEnv-vaccinated mice, there was a trend towards enhanced proliferation at all Ag doses in mice that also received the pRelA adjuvant when compared to unadjuvanted animals (Figure 3A). This trend was also observed in pGag-vaccinated animals where the overall stimulation index was higher when pRelA was co-delivered (Figure 3B). As well, in both Figure 3A,B, in addition to the overall stimulation index, fold increase graphs are included, with the ‘fold” value being a ratio of stimulation index of the pEnv + pRelA or pGag + pRelA groups divided by stimulation indexes of the pEnv or pGag alone groups. Thus, the stimulation index in pEnv and pGag vaccinated animals was increased by the inclusion of a pRelA adjuvant, at all vaccine doses tested. These responses were specific for the HIV Ags since minimal proliferation was observed in splenocytes from animals that received the pRelA adjuvant alone. Taken together, these results demonstrate that the pRelA DNA adjuvant enhances Ag-specific T cell proliferative responses against two individual specific antigens.

### 3.3. Enhanced Antibody Responses with Adjuvanted Vaccination

**Figure 4:**
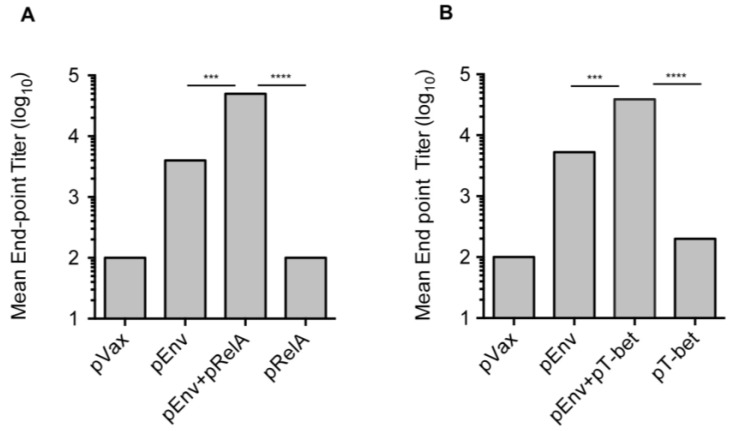
Improved B cell responses with pEnv vaccination and co-administered transcriptional molecular adjuvant. B cell/antibody responses were assessed in the sera of vaccinated mice (*n = 4*/group) seven days following the third immunization with pEnv alone, pEnv in combination with either pRelA or pTbet, each of the molecular adjuvants alone, or with empty vector control plasmid (pVax1). Anti-Env p120 antibody-binding titers were determined by ELISA. Data are presented as the mean endpoint titers. Statistically significant values are indicated; *** *p* < 0.001 (comparison between pEnv alone and pEnv + pRelA or pEnv + pT-bet) and **** *p* < 0.0001 (comparison between pRelA alone and pEnv + pRelA or pT-bet alone and pEnv + pT-bet).

Based on the observed adjuvant mediated increase in T cell IFN-γ and proliferative responses, the effects of these molecular adjuvants on B-cell induction was evaluated. HIV-1 Env-specific IgG was measured in the sera of vaccinated animals seven days following the third vaccination. As indicated, mice received pEnv either with or without co-administered pRelA or pTbet, pRelA or pTbet alone, or a pVax1 control plasmid (Figure 4). Measurable IgG responses were induced by pEnv alone at dilutions ranging from 1:50 to 1:500, but were non longer measurable at a dilution of 1:1000. Importantly, these responses were augmented at all dilutions by the inclusion of the pRelA or pTbet adjuvant when compared to the pEnv group alone. Specifically, differences were observed at the 1:50 sera dilution, where administration of pRelA and pTbet significantly enhanced the induction of HIV-1 Env-specific IgG responses (*p* = 0.0388 and *p* = 0.0062, respectively). Enhanced IgG responses were specific for Env since minimal antibody responses were observed in the sera from mice that were administered the pRelA or pTbet adjuvant alone. These data suggest that both transcription factor adjuvants elicited an enhanced humoral immune response that was analogous and consistent with the elevated IFN-γ levels and T cell proliferative responses observed following vaccination with pRelA or pTbet.

**Figure 5:**
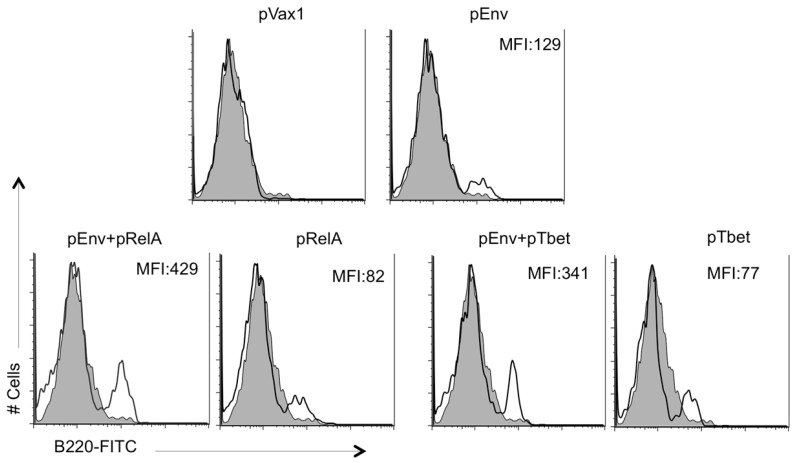
Molecular adjuvants enhance populations of B-cells at the site of immunization. Cells cultures from the muscle were analyzed by flow cytometry for expression of B220. The isolated cells were incubated in culture media for three days and these cells and then stained with DC subsets (CD11c^+^/CD11b^+^), B cells (B220^+^), T cells (CD4^+^ and CD8^+^ subsets), to distinguish monocytes/dendritic, B cells, T cells, respectively. Such differential staining allowed the exclusion of dendritic and T cells from subsequent analysis of B220 expression. Histograms show the B220^+^ expression on B cells exclusively using a specific mAb as well as an isotype-matched, irrelevant mAb as a control. The profile of an isotype-matched irrelevant Ab, used as a control (shaded area) is also indicated in the panels. MFI = mean fluorescent intensity which is proportional to the level of B220 expressing B cells.

One potential mechanism for the ability of the transcription factors to enhance antibody responses may be thorough increase in the number of activated B-cells. To access whether this was occurring, the pRelA administered muscle at the site of vaccination was biopsied three days after pEnv immunization with co-administrated pRelA followed by quantification of number of B220+ B-cells at the site of injection. The results indicated that pRelA and pEnv alone caused only a slight increase in B-cell trafficking to the site of injection compared to pVax1 administration alone (Figure 5). This is indicated by the MFI (mean fluorescent intensity) values shown in the individual FACS scans, which are directly proportional to the level of B220^+^ B cells. However, the addition of a pRelA adjuvant in combination with the pEnv vaccine further enhanced the number of B-cells at the site of injection.

## 4. Discussion

The study reported here is the first to report the ability of plasmid-expressed RelA and T-bet to function as molecular adjuvants for antigen specific DNA vaccine induced immunity. While the potential utility of RelA and T-bet are evident, the exact mechanism by which they enhance immunogenicity is currently unknown. For the RelA molecular adjuvant, it is likely that the over expression of this molecule directly drives NF-κB activation, much like many current adjuvants do indirectly. For example, many bacterial-derived carbohydrates such as those present in the GSK AS04 adjuvant and many veterinary adjuvants exert their pro-inflammatory effects through activating this pathway. Likewise, the recently approved MPL adjuvantTLR-4, which activates NF-κB and leads to enhanced T_h_1 and antibody responses. In the present study, we hypothesized that directly expressing RelA/p65 at the site of injection would potentially impact multiple cell types and multiple signaling pathways within a given cell resulting in NF-κB activation. This could lead to the stimulation of numerous pro-inflammatory signals for the induction of vaccine-specific T_h_1-type responses. NF-κB is expressed in all hematological cells and the pairing of the p65 subunit leads to multiple functional outcomes including activation. In this study, we also reported an approach to enhance the immunogenicity of DNA-based vaccines using T-bet as an adjuvant. Enhancement of antigen specific immune responses was confirmed by the fact that the T cell immune response was elicited in the T-bet co-administered mice compared to the pVax1 control. The enhanced immunity observed with molecular immune adjuvants may also have been the result of direct transfection of local DCs at the site of injection. It is known that the nuclear translocation of p65 is associated with the activation and maturation of DCs [61]. In addition, NF-kappa B activation controls the expression of critical co-stimulatory molecules such as CD80, CD86 and MHC class II along with pro-inflammatory cytokines such as IL-12, IL-6 and TNF-α [62,63]. Over expression of RelA in local DCs could likely increase cytokine production and co-stimulatory molecule expression, both of which could lead to improved T cell induction. Therefore, it is likely that the inclusion of a RelA-expressing molecular adjuvant could result in NF-kappa B activation in multiple cells at the site of injection, including resident DC and potentially could explain the improved T cell phenotype observed in this study.

In addition to stimulation of the T cell responses, enhanced antigen specific IgG production was also observed when a transcription adjuvant was used in combination with the vaccine. For the RelA adjuvant, NF-kappa B activation enhances the expression of adhesion molecules required for immune cell accumulation at the site of inflammation [64]. Further, studies in knockout mice suggest p65 is required for B-cell proliferation in response to BCR signaling [41]. Therefore, the inclusion of pRelA may facilitate the accumulation of Ag-specific cells at the site of vaccination. For the T-bet molecular adjuvant, *in vitro* over expression in B cell lines has been shown to result in Ab class switching, thereby increasing the level of IgG2a [65]. It is possible that IgG2a responses are enhanced herein since T-bet expression regulates the transcription of mature B cell receptors, which are necessary for the survival of memory B cells. In addition, the differential transcriptional stimulation determined by T-bet may regulate B cell potential for cytokine secretion, trafficking, and survival that ultimately permits flexibility in long-term Ag-specific immunoprotection [65]. Thus, data herein indicate that vaccination with co-expressed transcription factors associated with pro-inflammation and Th1 type development may contribute to the enhancement of vaccine-induced B cell responses.

Several viral and bacterial infection models have been tested extensively to define the transcription factors that may have a role in the development, differentiation and maturation of immune cells on particularly the CD8^+^ T-cell effector and memory populations [46]. Our results are in agreement with a model supporting a role for several transcriptional factors in the enhancement of primary antigen specific cellular and humoral responses. Overall, the work presented here suggests that pRelA and T-bet plays a significant role in creating the immune environment that influences the development and function of the strong vaccine induced CD8^+^ T-cell response and antibody production.

While the molecular adjuvanted E-DNA vaccine approaches presented herein have demonstrated the potential for increased Ag-specific immunity, it is important to consider possible safety issues that may be caused by transcription factor expression. Importantly, while increased NF-kappa B expression can lead to multiple immunological disorders and cancers [66,67] we observed no significant adverse safety events in mice vaccinated with the pRelA or pTbet molecular adjuvants. This may be explained by transient expression of these proteins by plasmid DNA that is known to persist for approximately 14–30 days. In addition, local expression of the plasmid DNA expressed transcription factors may help to drive immunity at the vaccination site while minimizing the possibility of systemic or off-target effects. Thus, it would be unlikely that a transcription factor-encoding genetic adjuvant would induce any serious long-term negative side effects. Furthermore, our group and others have studied extensively the use of various immune modulating plasmid-expressed adjuvants without any evidence for the development of adverse effects. This is an important safety consideration and demonstrates that administration of plasmid based transcriptional factors does not lead to global immune deregulation. However, future studies should further investigate the safety of the proposed adjuvants in preclinical studies utilizing nonhuman primates, before advancing to the clinic. In conclusion, this is the first report, to our knowledge, to demonstrate the ability of several transcription factors, when delivered as DNA expression plasmid, to enhance the immunogenicity of antigen specific DNA vaccines. 

## 5. Conclusions

The use of cellular transcription factors RelA and T-bet as molecular adjuvants for enhancing DNA vaccine-induced immunity was investigated in this report. When co-delivered along with a prototypical DNA vaccine by *in vivo* electroporation (EP), either of these putative adjuvants stimulated enhanced antigen-specific T and B cell responses as indicated by increased T cell numbers and IFN-γ production, as well as by an increase in antibody levels. This study builds on recent achievements demonstrating the potency of the enhanced DNA (E-DNA) vaccination method and establishes that transcription factors may serve as effective molecular adjuvants to boost vaccine-induced immunity.
